# Transdiagnostic inflammatory subgroups among psychiatric disorders and their relevance to role functioning: a nested case-control study of the ALSPAC cohort

**DOI:** 10.1038/s41398-022-02142-2

**Published:** 2022-09-09

**Authors:** Jonah F. Byrne, Colm Healy, David Mongan, Subash Raj Susai, Stan Zammit, Melanie Fӧcking, Mary Cannon, David R. Cotter

**Affiliations:** 1 Department of Psychiatry, Royal College of Surgeons in Ireland, Dublin, Ireland; 2 SFI FutureNeuro Research Centre, Royal College of Surgeons in Ireland, Dublin, Ireland; 3 Centre for Public Health, Queen’s University Belfast, Belfast, UK; 4 Centre for Academic Mental Health, Population Health Sciences, Bristol Medical School, University of Bristol, Bristol, UK; 5 Division of Psychological Medicine and Clinical Neurosciences, MRC Centre for Neuropsychiatric Genetics and Genomics, Cardiff University, Cardiff, UK

**Keywords:** Biomarkers, Neuroscience, Depression, Schizophrenia

## Abstract

Individuals with psychotic disorders and depressive disorder exhibit altered concentrations of peripheral inflammatory markers. It has been suggested that clinical trials of anti-inflammatory therapies for psychiatric disorders should stratify patients by their inflammatory profile. Hence, we investigated whether different subgroups of individuals exist across psychiatric disorders, based on their inflammatory biomarker signatures. We measured the plasma concentrations of 17 inflammatory markers and receptors in 380 participants with psychotic disorder, depressive disorder or generalised anxiety disorder and 399 controls without psychiatric symptoms from the ALSPAC cohort at age 24. We employed a semi-supervised clustering algorithm, which discriminates multiple clusters of psychiatric disorder cases from controls. The best fit was for a two-cluster model of participants with psychiatric disorders (Adjusted Rand Index (ARI) = 0.52 ± 0.01) based on the inflammatory markers. Permutation analysis indicated the stability of the clustering solution performed better than chance (ARI = 0.43 ± 0.11; *p* < 0.001), and the clusters explained the inflammatory marker data better than a Gaussian distribution (*p* = 0.021). Cluster 2 exhibited marked increases in sTNFR1/2, suPAR, sCD93 and sIL-2RA, compared to cluster 1. Participants in the cluster exhibiting higher inflammation were less likely to be in employment, education or training, indicating poorer role functioning. This study found evidence for a novel pattern of inflammatory markers specific to psychiatric disorders and strongly associated with a transdiagnostic measure of illness severity. sTNFR1/2, suPAR, sCD93 and sIL-2RA could be used to stratify clinical trials of anti-inflammatory therapies for psychiatric disorders.

## Introduction

Depressive disorder, first-episode psychosis (FEP), and generalised anxiety disorder have been associated with increased pro-inflammatory markers such as interleukin-6 (IL-6), tumour necrosis factor-alpha (TNF-α) or C-reactive protein (CRP) [1–5]. Several clinical trials of adjunctive anti-inflammatory therapies for psychotic disorders and depressive disorder have been conducted showing modest effect sizes [6–8]. There have been calls for future trials to stratify patients by their immune profile [6, 9] and for therapies to be guided by the understanding of transdiagnostic neurobiological mechanisms [10, 11].

Previous meta-analyses have assessed the variability of inflammatory markers in individuals with depression [12] and FEP [13] compared to controls. These studies found that the variability of IL-6, IL-8, IL-10, CRP, TNF-α and interferon-gamma (IFN-γ) is not higher in patients than controls. Indeed, there is evidence that CRP levels show less variability in individuals with depression [12] and that IL-6, IL-8 and TNF-α levels show less variability in individuals with psychotic disorders [13]. Less or equal variability of inflammatory markers in patients compared to controls does not support the hypothesis that inflammatory subgroups of psychotic disorder or depression exist, at least on the basis of individual markers.

Previous studies that have identified inflammatory subgroups based on multiple inflammatory markers have derived clusters containing both individuals with psychiatric disorders and controls [14–16], or have identified subgroups of individuals with depression or schizophrenia without direct inclusion of controls in the analysis [17–20]. In some cases, uncertainty remains as to whether identified clusters could also be found in a non-psychiatric population, thus potentially representing inflammatory processes that are not relevant to mental illness.

In this study, we aimed to investigate the existence of transdiagnostic inflammatory subgroups with specific relevance to psychiatric disorders, and to characterise such subgroups. Taking a data-driven approach, we applied a semi-supervised clustering method [21] that derives clusters of individuals with psychiatric disorders based on their differences from controls. Furthermore, the method allows for covariate adjustment and internal validation to estimate the stability of the clusters. In our analysis we included several cytokines, cytokine receptors and cellular adhesion molecules, as well as a putative biomarker of chronic inflammation; soluble urokinase plasminogen activation receptor [22, 23], and the complement-related inflammatory regulators; alpha-2-macroglobulin [24] and cluster of differentiation 93 (CD93) [25].

## Subjects and methods

### Participants and study design

The Avon Longitudinal Study of Parents and Children (ALSPAC) study is a prospective general population cohort [26–28]. Pregnant women in Avon, United Kingdom, with delivery dates between April 1, 1991, and December 31, 1992, were invited to participate, and 14,541 pregnancies were enroled. When the children were approximately 7 years old, an attempt was made to bolster the initial sample with children who did not join originally. The sample size at age 7 is 15,454 pregnancies of whom 14,901 children were alive at 1 year of age. Study data were collected and managed using REDCap electronic data capture tools [29]. The study website contains details of all data that are available through a fully searchable data dictionary and variable search tool (http://www.bristol.ac.uk/alspac/researchers/our-data).

Participants were invited to attend a clinic when they were approximately 24 years old. 4019 (40.4% of those invited) attended this clinic, where anthropometric measurements, questionnaires, and interviews were completed and blood samples were collected. This study is based on a subsample (*n* = 779) of individuals who attended this clinic.

Cases were defined as those meeting the criteria for diagnosis of either psychotic disorder, moderate/severe depressive disorder, or generalised anxiety disorder (*n* = 380) at age 24 years, as assessed by the Psychosis-Like Symptoms Interview [30, 31] or the revised Clinical Interview Schedule (CIS-R) [32]. All participants who met these criteria and concurrently provided consent for a plasma sample to be taken were included. Controls were defined as not meeting criteria for any of the above disorders (including mild depressive disorder) and not having suspected or definite psychotic experiences within the past year. Controls were sampled from participants who attended clinics at age 11, 17, and 24 and had data available for BMI (*n* = 399).

### Clinical measures

Psychiatric outcomes were defined as described previously [33].

#### Psychotic disorder and psychotic experiences

Participants completed the semi-structured Psychosis-Like Symptoms Interview (PLIKSi) to assess for psychotic symptoms [30, 31]. Trained interviewers assessed participants for psychotic experiences, including hallucinations, delusions, and experiences of thought interference. Psychotic experiences within the past year were rated by interviewers as not present, suspected or definitely present. Responses were coded according to the Schedules of Clinical Assessment in Neuropsychiatry [34]. As in previous studies [31, 35], psychotic disorder was defined as having at least one definite psychotic experience not attributable to sleep or fever, which recurred at least once per month over the previous six months, and was associated with severe distress, marked impairment of the participant’s social or occupational functioning, or led them to seek help.

#### Depressive disorder and generalised anxiety disorder

Participants completed the self-administered computerised version of the CIS-R [32]. In the CIS-R, the severity, frequency, and persistence of symptoms are assessed to derive diagnoses of depressive disorder (mild, moderate, or severe) and generalised anxiety disorder according to the International Classification of Diseases version 10 [36].

#### Clinical characteristics

As part of the CIS-R, participants reported on the presence of anhedonia and sleep problems. As part of the same clinic, participants reported if they were daily smokers, if they had any major physical health conditions, and whether they were not in employment, education or training (NEET; neither part-time nor full-time). Major physical health conditions included arthritis, diabetes, stroke, cancer, heart disease or heart problems. Data on medication use for hallucinations/delusions or other mental health problem were collected as part of the PLIKSi.

### Inflammatory biomarker measurements

Blood samples were collected and processed according to a standardised protocol. Plasma inflammatory biomarkers were measured using enzyme-linked immunosorbent assays (ELISA) and proximity extension assays (PEA) blind to case/control status. Samples were randomised according to a random sequence prior to biomarker measurements. Further details on sample collection and biomarker measurements can be found in the Supplementary methods.

#### Multiplex ELISA analytes

Plasma concentrations of IFN-γ, TNF-α, interleukin-1-beta (IL-1-β), IL-2, IL-4, IL-6, IL-8, IL-10, IL-13, CRP, soluble intracellular adhesion molecule-1 (ICAM-1) and soluble vascular cell adhesion molecule-1 (sVCAM-1) were measured using the multiplex V-Plex Pro-Inflammatory Panel 1 (Meso Scale Diagnostics (MSD; Maryland, USA), K15049D) and V-Plex Vascular Injury Panel 2 (MSD, K15198D) according to manufacturer’s instructions. Standards and samples were run in duplicate and the mean value for each duplicate pair was used in analyses. The lower limits of detection for each marker can be found in Supplementary Table 1.

#### Soluble urokinase plasminogen activation receptor (suPAR)

Plasma concentrations of suPAR were measured using the suPARnostic ELISA kit (Virogates, Birkerød, Denmark, https://www.virogates.com/suparnostic-elisa/) according to the manufacturer’s instructions. Standards were run in duplicate and samples in singlet. The lower limit of detection for this assay is 0.4 ng/ml.

#### Alpha-2-macroglobulin (A2M)

Plasma concentrations of A2M were measured using human alpha-2-macroglobulin ELISA kit (Abcam; Cambridge, UK; ab108888) according to manufacturer’s instructions. Standards were run in duplicate and samples in singlet. The lower limit of detection for this assay is 0.95 µg/ml.

#### Inflammatory biomarker receptors

Plasma concentrations of circulating tumour necrosis factor receptor 1 (TNFR1), tumour necrosis factor receptor 2 (TNFR2), interleukin-1 receptor type 1 (IL-1RT1), interleukin-1 receptor type 2 (IL-1RT2), interleukin-2 receptor subunit alpha (IL-2RA), interleukin-6 receptor subunit alpha (IL-6RA) and CD93 were measured using a commercially available PEA, Cardiovascular Panel III (Olink Proteomics; Uppsala, Sweden; https://www.olink.com). Samples were run in singlet. The lower limits of detection for each marker measured by PEA can be found in Supplementary Table 1.

The 7 receptors were chosen a priori from the 92 proteins available on the Cardiovascular Panel III. We hypothesised that the interactions between cytokines and cytokine receptors would be particularly informative. Referring to previous literature, we included cytokine receptors where the related cytokine was shown to have meta-analytical association with depression or psychotic disorders [1–4]. Due to previous evidence of the involvement of the complement system in psychosis [37–39], we included the complement-related protein CD93.

### Data pre-processing

As a means of quality control, inflammatory markers measured with multiplex ELISA were included in the analyses if ≥80% of plasma concentration values were above the limit of detection and ≥80% of concentration values had a coefficient of variation (CV) of ≤20%. Separate quality control criteria for suPAR, A2M and markers measured with PEA required ≥80% of plasma concentration values to be above the limit of detection.

Values below the limit of detection for any marker were replaced with the corresponding lower limit of detection divided by the square root of 2. Plasma sample concentration values were log-transformed where the Fisher-Pearson coefficient of skewness for the distribution of each marker was greater than 1. All values were subsequently converted to z-scores and winsorised within ±4 standard deviations to reduce the effects of outliers. Imputation was carried out using the K-Nearest Neighbours algorithm (KNN = 7). In total, 1.2% of all biomarker values were imputed, including values which were outside the range of the curve fit for each marker, values with a corresponding CV of >20% and values that were missing due to insufficient sample volume. The number of missing values for each marker is presented in Supplementary Table 2.

### Subtyping

We applied the semi-supervised clustering algorithm HYDRA (heterogeneity through discriminant analysis) [21] to the inflammatory biomarker data of the participants to investigate the presence of stable, reproducible clusters. The algorithm HYDRA fits multiple linear classifiers, which each distinguish clusters of cases from controls for separate reasons. The number of clusters (*K*) being evaluated is directly related to the number of linear classifiers used. Disorder clusters are thus identified by the shared characteristics that delineate them from controls. In this study, cases are individuals with psychiatric disorders, and the characteristics used to separate cases from controls are patterns of inflammatory marker levels.

The semi-supervised approach of HYDRA has advantages over other clustering methods: by using controls as a reference population, the algorithm clusters cases based on features relevant to the disorder of interest. Furthermore, it allows correction for covariates (in this case, sex and BMI), in attempt to capture heterogeneity related to the disorders and minimise confounding to the clustering solution.

A “hold-out” internal validation strategy was implemented using 100 stratified subsamples, to improve the potential generalisability of our results and minimise the effect of outliers. Each subsample consisted of a random sample of 80% of the cohort with preserved proportions of cases and controls. Clustering was carried out on each of the subsamples and a consensus solution across subsamples was determined. Solutions between 2–5 clusters were tested. We limited our analysis to a 5-cluster solution as we decided a priori that solutions with more than 5 clusters would lack clinical utility. The stability of the clustering solutions was assessed by the mean Adjusted Rand Index (ARI) across subsamples, where a higher ARI indicates greater cluster stability. Further details on HYDRA are found in the Supplementary methods.

As the clusters derived in this study differed in proportions of daily smokers, we investigated whether the clusters were confounded by smoking. We repeated our analysis adjusting for a binary variable of daily smoker (Yes/No) in addition to sex and BMI.

### Statistical analysis

The significance of clustering solutions was assessed using the SigClust method [40, 41], with the null hypothesis that the data are from a single Gaussian distribution. The parameters for the null Gaussian distribution are estimated from the data, and the significance of clustering is then tested by simulation from this distribution. The “SigClust” package for R was run with 1000 simulations.

Additionally, a null distribution was derived by random permutation analysis, which indicated the stability of clustering solutions that could be found by chance (further details in Supplementary methods). Welch’s *t*-test for unequal variances was used to compare repetitions of the main analysis to the null distribution.

Currently, there are no guidelines for estimating the sample-size requirements for the clustering method implemented in this study. To reduce the risk of overfitting, we kept the number of events-per-variable above 20.

Differences in case and control characteristics or cluster characteristics were compared using the Wilcoxon–Mann–Whitney two-sample rank sum test (two-sided) or Pearson’s Chi-squared test. We chose to investigate differences in potential confounders between clusters (sex, BMI, smoking, sleep problems, major physical health conditions, medication use for mental health problems). Furthermore, we investigated differences in the major psychiatric symptoms; anhedonia and psychotic experiences, which have previously been associated with inflammatory markers [20, 42, 43] and differences in role functioning between clusters, as measured by NEET status. P-values were not adjusted for multiple comparisons.

Logistic regression models were used to investigate whether clinical characteristics were associated with cluster membership beyond certain confounders, using binary cluster membership as the dependent variable and the clinical characteristic as the independent variable. The analyses were conducted adjusting for sex, BMI, daily smoking and medication use for mental health problems. Using the same approach, logistic regression models were also used to investigate whether each inflammatory marker was associated with cluster membership beyond certain confounders.

Analyses were conducted in R (4.1.0) and python (3.9).

## Results

### General descriptive statistics

In our sample of psychiatric disorder cases, 40 participants (10.5% of cases) met criteria for psychotic disorder, 202 (53.1%) met criteria for moderate or severe depression and 268 (70.5%) met criteria for GAD at age 24 years. 113 (29.7% of cases) participants met criteria for more than one disorder.

Participants who met the criteria for any of the three psychiatric disorders had a higher proportion of females (74.5%) than the control population (52.4%; *p* < 0.001) and had a higher mean BMI (25.4 kg/m^2^; 95% confidence interval [CI] 24.8–26.0 kg/m^2^) than controls (24.0 kg/m^2^; 95% CI 24.4–23.6 kg/m^2^; *p* = 0.018) (Table 1).

**Table 1:** Characteristics of the study cohort.

	Psychiatric disorder	Controls	*P* value
	(*n* = 380)	(*n* = 399)	
Age in years, mean (SD)	24.1 (0.8)	24.0 (0.8)	0.353
BMI in kg/mg^2^, mean (SD)	25.4 (6.0)	24.1 (4.3)	0.018
Sex, *n* (%)			
Female	283 (74.5%)	209 (52.4%)	<0.001
Male	97 (25.5%)	190 (47.6%)	
Psychotic disorder, *n* (%)	40 (10.5%)	N/A	N/A
Moderate/severe depressive disorder, *n* (%)	202 (53.1%)	N/A	N/A
Generalised anxiety disorder, *n* (%)	250 (70.5%)	N/A	N/A
>1 psychiatric disorder, *n* (%)	113 (29.7%)	N/A	N/A
Has received medication for hallucinations/delusions or other mental health problem, *n* (%)	82 (21.6%)	N/A	N/A

### Cluster analysis

**Fig. 1:**
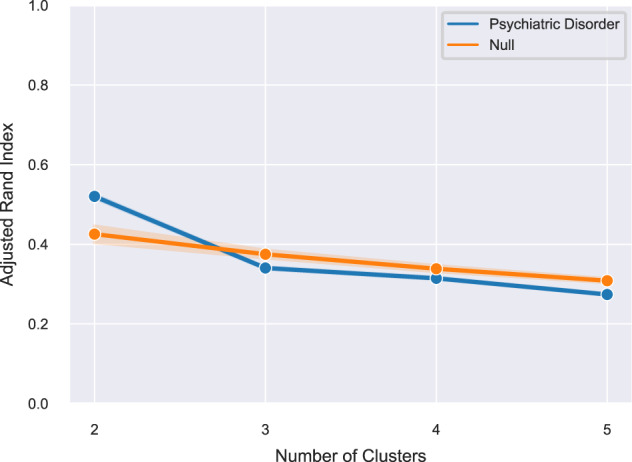
The Adjusted Rand Index for *K* clusters of the psychiatric disorder group compared to the null distribution obtained from random permutations. Data are represented as mean ±95% CI for each clustering solution between 2 and 5 clusters. The highest ARI was for a two-cluster solution of individuals with psychiatric disorders.

The markers IL-6, IL-8, IL-10, TNF-α, IFN-γ, CRP, sVCAM-1, sICAM-1, suPAR, A2M, IL-1RT1, IL-1RT2, IL-2RA, IL-6RA, TNFR1, TNFR2 and CD93 met quality control criteria and were included in the analysis. HYDRA was run with internal validation across 100 subsamples. The highest ARI (0.52; standard deviation (SD) = 0.01) was for a two-cluster solution of psychiatric disorder participants (Fig. 1). Simulation with the Sigclust method indicated that the clusters explained the data better than a Gaussian distribution (*p* = 0.021). Comparison with results from permutation analyses (ARI of 0.43 ± 0.11 for two clusters) indicated the stability of the clustering solution performed better than chance (*p* < 0.001).

**Fig. 2:**
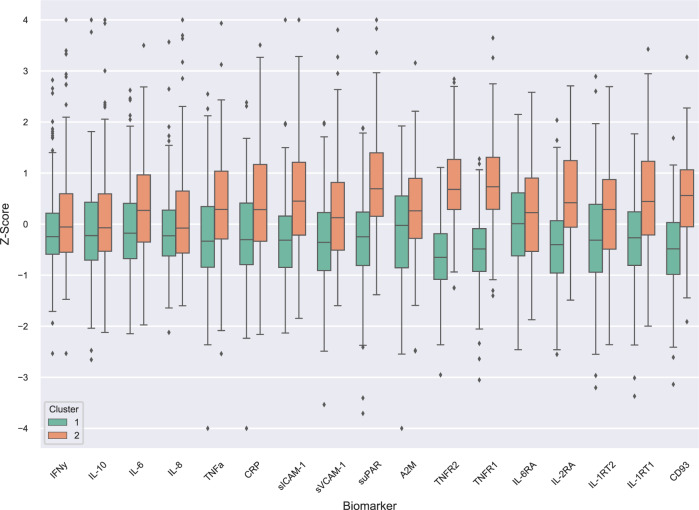
Distribution of standardised inflammatory biomarker values in each cluster as determined by semi-supervised clustering. A consensus clustering solution across 100 subsamples was determined by the algorithm HYDRA, adjusting for sex and BMI. Simulation with the Sigclust method indicated that the clusters explained the data better than a Gaussian distribution (*p* = 0.021). Interferon-gamma (IFN-γ), interleukin-10 (IL-10), interleukin-6 (IL-6), interleukin-8 (IL-8), tumour necrosis factor-alpha (TNF-α), C-reactive protein (CRP), soluble intracellular adhesion molecule-1s (ICAM-1), soluble vascular cell adhesion molecule-1 (sVCAM-1), soluble urokinase plasminogen activation receptor (suPAR), alpha-2-macroglobulin (A2M), tumour necrosis factor receptor 1 (TNFR1), tumour necrosis factor receptor 2 (TNFR2), interleukin-1 receptor type 1 (IL-1RT1), interleukin-1 receptor type 2 (IL-1RT2), interleukin-2 receptor subunit alpha (IL-2RA), interleukin-6 receptor subunit alpha (IL-6RA), cluster of differentiation 93 (CD93).

The 95% CIs for differences in cluster inflammatory marker means indicated cluster 2 had broadly higher levels of inflammatory markers than cluster 1. The largest differences between the clusters were observed for the markers TNFR2, TNFR1, suPAR, CD93 and IL2RA (Fig. 2 and Table 2). Differences in inflammatory marker levels between each cluster and controls are presented in Supplementary Table 6. Cluster 2 had broadly higher levels of inflammatory markers than controls and cluster 1 had broadly lower levels of inflammatory markers than controls. Cluster 2 inflammatory marker levels were separated from controls more strongly than cluster 1 levels were separated from controls. The clusters differed in proportions of individuals who were daily smokers, had psychotic experiences within the past year and were not in employment, education or training (Table 3).

**Table 2:** Difference in cluster inflammatory marker means, 95% CI.

	Difference in means	95% CI	
IFN-γ	0.26	0.07	0.46
IL-10	0.29	0.08	0.50
IL-6RA	0.30	0.11	0.50
IL-8	0.35	0.15	0.56
A2M	0.40	0.20	0.59
IL-6	0.44	0.25	0.63
sVCAM-1	0.52	0.32	0.72
IL-1RT2	0.55	0.35	0.75
TNF-α	0.58	0.38	0.79
CRP	0.60	0.40	0.81
IL-1RT1	0.74	0.55	0.93
sICAM-1	0.79	0.59	0.99
IL-2RA	1.01	0.84	1.18
CD93	1.03	0.86	1.20
suPAR	1.15	0.96	1.34
TNFR1	1.31	1.15	1.48
TNFR2	1.47	1.32	1.62

Difference in means of normalised inflammatory markers between cluster 1 and cluster 2 are presented using cluster 1 as the reference group.

**Table 3:** Characteristics of the transdiagnostic clusters.

		Cluster 1	Cluster 2	*P* value
		(*n* = 217)	(*n* = 163)	
Psychotic disorder, *n* (%)		18 (8.3%)	22 (13.5%)	0.143
Depressive disorder, *n* (%)		113 (52.1%)	89 (54.6%)	0.700
Generalised anxiety disorder, *n* (%)		157 (72.4%)	111 (68.1%)	0.432
Sex	Male, *n* (%)	58 (26.7%)	39 (23.9%)	0.616
	Female, *n* (%)	159 (73.3%)	124 (76.1%)	
BMI, mean (SD)		24.6 (4.9)	26.3 (7.0)	0.049
Daily smoker, *n* (%)		35 (16.1%)	45 (27.6%)	0.007
Major physical health condition, *n* (%)		5 (2.3%)	<5 (<3.1%)^a^	0.924
Number of nights with sleep problems in past 7 nights, *n* (%)	1–3	76 (35.0%)	62 (38.0%)	0.693
	4+	67 (30.9%)	44 (27.0%)	
Has received medication for hallucinations/delusions or other mental health problem, *n* (%)		39 (18.0%)	42 (25.8%)	0.087
Suspected or definite psychotic experiences within the past year, *n* (%)		34 (15.7%)	44 (27.0%)	0.010
Anhedonia within the past month	Less enjoyment than usual, *n* (%)	144 (66.4%)	98 (60.1%)	0.106
	Did not enjoy anything, *n* (%)	8 (3.7%)	14 (8.6%)	
Not in employment, education or training		18 (8.3%)	35 (21.5%)	<0.001

^a^Data suppressed due to small cell counts.

Logistic regression analyses demonstrated that the association between NEET status and cluster membership remained while adjusting for sex, BMI, daily smoking and medication use for mental health problems (odds ratio [OR] 2.80, 95% CI 1.50, 5.35). The association between psychotic experiences and cluster membership attenuated while adjusting for the same covariates (OR 1.80, 95% CI 0.99, 3.30).

### Sensitivity analyses

To investigate whether the clustering solution was confounded by smoking, we conducted a sensitivity analysis where the algorithm HYDRA was run with daily smoker as an additional covariate. The highest ARI was for a two-cluster solution (mean = 0.50, SD = 0.01). The inflammatory marker distributions of the two clusters (Supplementary Fig. 2) were in agreement with our main analysis. Simulation with the Sigclust method indicated that the clusters explained the data better than a Gaussian distribution (*p* = 0.039). Comparison with results from permutation analyses (ARI of 0.41 ± 0.12 for two clusters) indicated the stability of the clustering solution performed better than chance (*p* = 0.018) while adjusting for smoking.

In addition, we conducted a sensitivity analysis where the algorithm HYDRA was run excluding individuals with a major physical health condition or BMI > 30. The highest ARI (0.50, SD = 0.02) was for a two-cluster solution. Simulation with the Sigclust method indicated that the clusters explained the data better than a Gaussian distribution (*p* = 0.017). The inflammatory marker distributions of the clusters obtained in this sensitivity analysis were in agreement with our main analysis (Supplementary Fig. 3).

## Discussion

In a large population-based cohort, we investigated the existence of inflammatory subgroups using a transdiagnostic approach and data-driven, multivariable methods that had the potential to reveal inflammatory subgroups with specific relevance to psychiatric disorders.

Our analysis identified two subgroups with fair stability (ARI = 0.52 ± 0.01). Thus, we found evidence for inflammatory subgroups of psychiatric disorders. This is in contrast to evidence from previous meta-analyses which did not find that the variability of cytokines was greater in patients than in controls [12, 13]. These previous studies by Osimo et al. and Pillinger et al. investigated evidence for subgroups of depression [12] or FEP [13] alone based on individual inflammatory cytokines, while the current study assessed 17 inflammatory markers and searched for subgroups among 3 psychiatric disorders. Furthermore, the largest differences observed between the subgroups we identified were for levels of inflammatory marker receptors that could were not examined by Osimo et al. and Pillinger et al., rather than inflammatory markers such as IL-6 and CRP, which have been a primary focus of immunopsychiatry research.

Among the strongest predictors of the two subgroups we have described are two TNF receptors, (soluble TNFR1, TNFR2) and the soluble alpha-subunit of the IL-2 receptor, IL2RA. sTNFR1/2 are thought to regulate inflammatory signalling by TNF-α by competing with membrane-associated TNF receptors for TNF-α [44]. It has been previously argued that receptors such as sTNFR1 may be a better indicator of the TNF-α system activity than TNF-α itself due to difficulties in the reliable measurement of TNF-α [45–47]. sIL-2RA is used as a marker of T-cell activation and is increased in several cancers and in multiple sclerosis [48, 49]. The clusters also differentiated strongly on levels of sCD93 and suPAR. CD93 is expressed on microglia and is involved in central nervous system (CNS) inflammation [25, 50, 51]. However, the degree to which peripheral CD93 reflects CNS inflammation has not yet been determined. suPAR has been identified as a predictor of chronic diseases [52], disease severity and mortality [53, 54] and is thus regarded as a measure of chronic inflammation [22, 23]. Elevated levels of TNFR1/2, CD93 and suPAR levels may reflect a unique pattern of inflammation that is particularly relevant to a subgroup of individuals with psychiatric disorders.

The subgroups we identified are of potential transdiagnostic clinical significance. In this study, the subgroup with higher levels of inflammatory markers had a greater proportion of individuals who had psychotic experiences in the past year and a greater proportion of individuals who were not in employment, education or training, the latter finding being the strongest. Social and role functioning are increasingly recognised as important outcomes [55–59], and psychiatric disorders have been associated with future long-term exclusion from education, employment or training while adjusting for sociodemographic confounders [60]. In line with this, it has previously been highlighted that those with psychotic experiences represent the more severe end of a continuum of ‘common mental distress’ [61]. Our results indicate the subgroups we identified are relevant to potential transdiagnostic measures of illness impairment or severity; NEET status and the presence of psychotic experiences. The subgroup with higher levels of inflammatory markers also had a greater proportion of individuals who were daily smokers. As tobacco smoking can increase peripheral inflammatory marker levels [62, 63], we conducted an additional analysis including daily smoking additional binary covariate. Including the smoking covariate had little effect on our results, suggesting that the inflammatory subgroups were not confounded by smoking status. Furthermore, there was no evidence that the clusters differed in the proportions of individuals with major physical health conditions or sleep problems.

The methods we applied and the cross-sectional nature of the data in this study do not allow us to infer causality. While we were able to adjust for confounders such as sex, BMI and smoking (additionally, participants were all of a similar age), the clustering solutions we have described could reflect differences in lifestyle factors such as diet and physical activity, as well as differences in socio-economic status [62, 63]. Alternatively, the clusters could reflect a subgroup of individuals with long-standing chronic inflammation (as indicated by higher levels of suPAR [22, 23]) that is associated with their poorer role functioning (as indicated by the higher proportions of NEET status in cluster 2).

The subgroups we identified differed most prominently in levels of soluble TNF-α receptor 1 and 2. A previous randomised-controlled trial (RCT) in individuals with treatment-resistant depression investigated the efficacy of infliximab, a monoclonal antibody for TNF-α, in the reduction of depressive symptoms [64]. No overall effect of infliximab over placebo was found, although a significant interaction between treatment, time and baseline CRP concentration with reduction in depressive symptom score was found. There was no such interaction between treatment, time and baseline levels of sTNFR1/2. A separate RCT of adjunctive infliximab in bipolar depression also reported absence of a sustained main effect of infliximab over placebo on anhedonia. However, the authors reported an interaction between treatment, time and baseline sTNFR1 with reduction in anhedonia [65]. In the present study, subgroup membership was not solely based on levels of sTNFR1/2, with circulating levels of suPAR, IL-2R and CD93 also differentiating strongly between the subgroups. These five markers together may confer additional information beyond sTNFR1/2 alone and could be used in the design of targeted trials of infliximab, other immune-regulating therapeutics, or exercise-based interventions [66, 67] for individuals with psychiatric disorders.

Recently there have been alternative approaches to ensuring specificity of clustering solutions to psychiatric populations. A previous study found evidence for a subgroup of patients with schizophrenia and bipolar disorder positive for the human endogenous retrovirus type W (HERV-W) envelope protein that exhibit increased levels of IL-6 and IL-1β [68]. In this case, the addition of the HERV-W biomarker aided in the identification of inflammatory subgroups specific to the psychotic disorders. A recent study by Luo et al. [69] clustered schizophrenia patients by their epigenetic profile and identified two subgroups that differed in leucocyte cell counts and neuroanatomical features. Similar subgroups were found when repeating the clustering with controls only, however, the control subgroups did not show neuroanatomical differences, demonstrating specificity of the patient clusters to schizophrenia.

This study has a number of strengths and limitations. Firstly, this study benefitted from a large subsample of a well-characterised general population cohort. However, the ALSPAC sample is subject to attrition, which may affect the generalisability of these results. Attrition in ALSPAC differs along the socio-economic gradient and there may be individuals with lower levels of functioning that have not been represented in this study. In our study, 30% of participants with one psychiatric disorder also met criteria for another psychiatric disorder, supporting the transdiagnostic approach to our research question. However, while we were able to include large numbers of individuals with depressive disorder and GAD, our study included relatively few individuals with psychotic disorder (*n* = 40) which limits the applicability of our results to psychotic disorder alone. Furthermore, there are no specific guidelines currently available for sample size requirements of the clustering method used, and our study would have benefitted from an even larger sample. The lack of an external validation sample is also a limitation of this study. Our analysis should be repeated in another sample to investigate whether the clustering solutions are reproducible.

The extent to which cytokines can be measured reliably with multiplex immunoassays varies considerably [70]. While some inflammatory markers, such as IL-1β and IL-2, did not pass our quality control, we were able to include a wide range of peripheral biomarkers in our analyses as well as receptors for IL-1β and IL-2. Our findings that inflammatory marker receptors may be superior predictors of inflammatory subtypes in psychiatric disorders may simply be a reflection of the differences in sensitivity between the two methods used: multiplex ELISA and PEA. Finally, the measures of anhedonia and role functioning in this study were relatively crude. Continuous measures of the severity of anhedonia and role functioning (capturing the amount of support needed in the current role) may have been more informative.

To investigate the existence of inflammatory subgroups specific to psychiatric disorders, we have used a method that has three clear advantages: (i) controls are used as a reference population, (ii) covariate adjustment is possible, and (iii) the stability of clusters can be assessed through internal validation techniques as an initial measure of their potential reproducibility. The algorithm HYDRA has previously been evaluated on genetic data [21] and has indicated the existence of two neuroanatomical clusters of schizophrenia using structural MRI data [41, 71, 72]. Here, we have applied the algorithm to peripheral inflammatory marker data, allowing us to consider the relevance of the clustering solutions to psychiatric disorders in light of data from controls, and adjust for the confounders sex and BMI [62]. We believe the subgroups we have identified will have better chances of being reproducible and applicable due to the advantages of the methods we have applied. However, it should be acknowledged that our modelling strategy makes the assumption that there are inflammatory subgroups specific to individuals with psychiatric disorders. In an alternative model, one could assume that there are inflammatory subgroups among the general population, with certain subgroups having greater risk of psychiatric disorder or symptoms. Nevertheless, the present study provides evidence for the former model.

In conclusion, this study found evidence for the presence of two transdiagnostic inflammatory subgroups of individuals with psychiatric disorders, which showed differences in their role functioning. These findings may be particularly useful in the identification of subgroups that could be targeted in clinical trials of anti-inflammatory therapeutics or interventions. We aim to replicate our results in future studies using a reduced panel of inflammatory biomarkers.

## Supplementary information

Supplemental Material

## Data Availability

The code used to generate the results in this study are available upon reasonable request from the corresponding author.
