# Supplementary material for: Transdiagnostic inflammatory subgroups among psychiatric disorders and their relevance to role functioning: a nested case-control study of the ALSPAC cohort
Source: Transl Psychiatry. 2022 Sep 9;12:377. doi: 10.1038/s41398-022-02142-2 (PMC9463145; doi:10.1038/s41398-022-02142-2)
Supplement: Supplementary file 1 — Supplemental Material [file 41398_2022_2142_MOESM1_ESM.docx]

[Supplementary methods: 2](#_Toc108710440)

[Supplementary tables: 7](#_Toc108710441)

[Supplementary table 1: Lower limits of detection for inflammatory markers measured with multiplex ELISA and PEA. 7](#_Toc108710442)

[Supplementary table 2: Number of missing values for inflammatory markers. 8](#_Toc108710443)

[Supplementary table 3. Coefficients of variation for plasma inflammatory markers measured with ELISA. 9](#_Toc108710444)

[Supplementary table 4. Coefficients of variation for PEA experiments. 10](#_Toc108710445)

[Supplementary table 5: Characteristics of the transdiagnostic clusters with missing data counts: 11](#_Toc108710446)

[Supplementary table 6: Difference in inflammatory marker means between clusters and controls, 95% CI. 12](#_Toc108710447)

[Supplementary table 7: Odds ratios from adjusted logistic regression analyses examining the association between inflammatory biomarker levels and cluster membership from the main analysis. 13](#_Toc108710448)

[Supplementary table 8: The relationship between clinical variables and each standardised biomarker. 14](#_Toc108710449)

[Supplementary figures: 15](#_Toc108710450)

[Supplementary figure 1: Distribution of standardised inflammatory biomarker values in each cluster as determined in the main analysis. 15](#_Toc108710451)

[Supplementary figure 2: Distribution of standardised inflammatory biomarker values in each cluster as determined in the sensitivity analysis adjusting for daily smoking. 16](#_Toc108710452)

[Supplementary figure 3: Distribution of standardised inflammatory biomarker values in each cluster as determined in the sensitivity analysis excluding individuals with chronic inflammatory disorders or BMI >30. 17](#_Toc108710453)

[Supplementary figure 4: Distribution of standardised inflammatory biomarker values in each cluster as determined in the sensitivity analysis excluding individuals who fasted for less than 8 hours before their blood sample was taken. 18](#_Toc108710454)

[Supplementary figure 5: Distribution of standardised inflammatory biomarker values in each cluster as determined in the sensitivity analysis adjusting for PEA experiment. 19](#_Toc108710455)

[Supplementary figure 6: Depiction of semi-supervised clustering with HYDRA. 20](#_Toc108710456)

[Supplementary results: 21](#_Toc108710457)

[References 22](#_Toc108710458)

Supplementary methods:

**Sample collection**

Blood samples were collected according to a standardised protocol. Participants were requested to fast during the 6 hours prior to attending the clinic. Samples were obtained between 8am and 2pm in >99% of individuals. Following collection, plasma was separated by centrifugation and stored at −80°C. Samples underwent one freeze-thaw cycle for aliquoting prior to analysis. The range of storage time between plasma collection and biomarker measurement was 0.3 – 2.7 years.

**Inflammatory biomarker measurements**

*Multiplex ELISA analytes:* Plasma concentrations of interferon gamma (IFN-γ), tumour necrosis factor alpha (TNF-α), interleukin-1-beta (IL1-β), IL-2, IL-4, IL-6, IL-8, IL-10, and IL-13 were measured using the multiplex V-Plex Pro-Inflammatory Panel 1 Human kit (Meso Scale Diagnostics (MSD; Maryland, USA), catalogue number K15049D); C-reactive protein (CRP), soluble intracellular adhesion molecule-1 (ICAM-1) and soluble vascular cell adhesion molecule-1 (sVCAM-1) were measured using the multiplex V-Plex Vascular Injury Panel 2 Human kit (MSD, catalogue number K15198D) according to manufacturer’s instructions. Optical densities were measured using a Sector Imager 2400 microplate reader (MSD). For each plate, a standard curve was generated, and the plasma concentrations of each marker were interpolated using the MSD Workbench software. Standards and samples were run in duplicate and the mean value for each duplicate pair was used in analyses. The coefficient of variation of samples was calculated using the optical density measurements of technical replicates. The lower limits of detection for each marker can be found in Supplementary Table 1.

*Soluble urokinase plasminogen activation receptor (suPAR):* Plasma concentrations of suPAR were measured using the suPARnostic ELISA kit (Virogates, https://www.virogates.com/suparnostic-elisa/) according to the manufacturer’s instructions. Optical densities were measured using a SpectraMax M3 microplate reader. For each plate, a standard curve was generated and plasma concentrations were interpolated using Virogates’ custom results calculation tool (available at <https://www.virogates.com/support>). Standards were run in duplicate and samples in singlet. The lower limit of detection for this assay is 0.4 ng/ml.

*Alpha-2-macroglobulin (A2M):* Plasma concentrations of A2M were measured using human alpha-2-macroglobulin ELISA kit (Abcam, ab108888) according to manufacturer’s instructions. Optical densities were measured using a SpectraMax M3 microplate reader. For each plate, a standard curve was generated and plasma concentrations were interpolated using GraphPad Prism 8 software. In line with the manufacturer’s recommendations, a four-parameter logistic curve was fit. Standards were run in duplicate and samples in singlet. The lower limit of detection for this assay is 0.95 µg/ml.

*Inflammatory biomarker receptors:* Plasma concentrations of circulating tumour necrosis factor receptor 1 (TNF-R1), tumour necrosis factor receptor 2 (TNF-R2), interleukin-1 receptor type 1 (IL-1RT1), interleukin-1 receptor type 2 (IL-1RT2), interleukin-2 receptor subunit alpha (IL-2RA), interleukin-6 receptor subunit alpha (IL-6RA) and cluster of differentiation 93 (CD93) were measured using a commercially available proximity extension assay (PEA), Cardiovascular Panel III (Olink Proteomics; <https://www.olink.com>). These 7 receptors were chosen *a priori* from the 92 proteins available on the Cardiovascular Panel III. We hypothesised that the interactions between cytokines and cytokine receptors and the complement-related proteins CD93 and A2M would be particularly informative. Samples were run in singlet. The lower limits of detection for each marker measured by PEA can be found in Supplementary Table 1.

**Data availability**

Access to ALSPAC data can be requested by researchers through an online proposal system. Information regarding access can be found on the ALSPAC website (<http://www.bristol.ac.uk/media-library/sites/alspac/documents/researchers/data-access/ALSPAC_Access_Policy.pdf>).

**PEA data normalisation:**

The samples in this study were spread across two separate PEA experiments. As recommended by Olink, ten samples were included in both experiments and were later used as bridging samples for normalisation between the experiments (<https://www.olink.com/application/data-normalization-and-standardization>). Bridge normalisation was carried out in the OlinkAnalyze package for R. As a sensitivity analysis we repeated the clustering with HYDRA adjusting for PEA experiment number.

**Pre-processing of covariates:**

There were no missing values for age or sex. Nine missing BMI values (2.4% of participants with psychiatric disorders) were imputed using K-Nearest Neighbours (*K* = 7) using height, weight and BMI at age 18 as auxiliary variables. All other clinical characteristics had <3.2% missing data (Supplementary table 5). Missing values for these variables were replaced with the most frequent category.

**HYDRA:**

HYDRA [1] performs classification and clustering simultaneously. It separates cases from healthy controls by fitting multiple linear maximum-margin classifiers or hyperplanes. Together, these hyperplanes form a shape called a convex polytope which divides cases and controls. Patient subtypes are derived from the association of groups of cases with individual hyperplanes (Supplementary figure 4).

A “hold-out” cross-validation strategy was used, where each cross-validation subsample consists of a random sample of 80% of the cohort with preserved proportions of cases and controls. Clustering is run on each of the subsamples and a consensus solution across subsamples is determined. The Adjusted Rand Index (ARI) [2] is calculated between clustering solutions from pairs of subsamples, measuring their agreement. The mean ARI across the pairs represents the stability of the final clustering solution.

The python implementation of HYDRA (pyHYDRA version 1.0.8, now contained within Machine Learning for Neuroimaging (MLNI; <https://github.com/anbai106/mlni>), was used in python (version 3.6). We ran HYDRA with the following parameters:

Initialisation strategy: Determinantal point processes (DPP); Iterations to optimise the polytope: 50; Number of repeats for consensus clustering: 20; Regularisation parameter: 0.25; Number of cross-validation folds (“hold-out” strategy): 100.

**Permutation analysis:**

In line with previous implementation of HYDRA [3] a null distribution of clustering stability was determined through the random labelling of controls. Controls were randomly labelled with case and control status in the same proportion as the actual experiment, and HYDRA was run as described previously. This procedure was repeated 100 times to obtain a null distribution of ARIs. The actual experiment was repeated 20 times to obtain a distribution, and the two distributions were then compared. For the sensitivity analysis adjusting for daily smoking, the actual experiment was repeated 10 times and the permutation analysis was repeated for 50 randomly-labelled control datasets.

Supplementary tables:

Supplementary table 1: Lower limits of detection for inflammatory markers measured with multiplex ELISA and PEA.

| **Inflammatory Marker** | **Concentration (pg/mL)** |
| --- | --- |
| IFN-γ | 0.37 |
| IL-1β | 0.05 |
| IL-2 | 0.09 |
| IL-4 | 0.02 |
| IL-6 | 0.06 |
| IL-8 | 0.07 |
| IL-10 | 0.04 |
| IL-12p70 | 0.11 |
| IL-13 | 0.24 |
| TNF-α | 0.04 |
| CRP | 1.33 |
| VCAM-1 | 6.00 |
| ICAM-1 | 1.94 |
| TNFR2 | 1.91 |
| TNFR1 | 3.81 |
| IL-6R | 0.12 |
| IL-2R | 0.06 |
| IL-1RT1 | 0.03 |
| IL-1RT2 | 0.95 |
| CD93 | 0.95 |

Interferon gamma (IFN-γ), interleukin-10 (IL-10), interleukin-6 (IL-6), interleukin-8 (IL-8), tumour necrosis factor alpha (TNF-α), C-reactive protein (CRP), soluble intracellular adhesion molecule-1s (ICAM-1), soluble vascular cell adhesion molecule-1 (sVCAM-1), soluble urokinase plasminogen activation receptor (suPAR), alpha-2-macroglobulin (A2M), tumour necrosis factor receptor 1 (TNF-R1), tumour necrosis factor receptor 2 (TNF-R2), interleukin-1 receptor type 1 (IL-1RT1), interleukin-1 receptor type 2 (IL-1RT2), interleukin-2 receptor subunit alpha (IL-2RA), interleukin-6 receptor subunit alpha (IL-6RA), cluster of differentiation 93 (CD93).

Supplementary table 2: Number of missing values for inflammatory markers.

| **Inflammatory Marker** | ***n* missing values** |
| --- | --- |
| IFN-γ | 37 |
| IL-6 | 14 |
| IL-8 | 5 |
| IL-10 | 11 |
| TNF-α | <5 |
| CRP | 10 |
| VCAM-1 | 10 |
| ICAM-1 | 27 |
| suPAR | 0 |
| A2M | <5 |
| TNFR2 | 6 |
| TNFR1 | 6 |
| IL-6R | 5 |
| IL-2R | 5 |
| IL-1RT1 | 5 |
| IL-1RT2 | 5 |
| CD93 | 6 |

Data supressed due to small cell counts. Interferon gamma (IFN-γ), interleukin-10 (IL-10), interleukin-6 (IL-6), interleukin-8 (IL-8), tumour necrosis factor alpha (TNF-α), C-reactive protein (CRP), soluble intracellular adhesion molecule-1s (ICAM-1), soluble vascular cell adhesion molecule-1 (sVCAM-1), soluble urokinase plasminogen activation receptor (suPAR), alpha-2-macroglobulin (A2M), tumour necrosis factor receptor 1 (TNF-R1), tumour necrosis factor receptor 2 (TNF-R2), interleukin-1 receptor type 1 (IL-1RT1), interleukin-1 receptor type 2 (IL-1RT2), interleukin-2 receptor subunit alpha (IL-2RA), interleukin-6 receptor subunit alpha (IL-6RA), cluster of differentiation 93 (CD93).

Supplementary table 3. Coefficients of variation for plasma inflammatory markers measured with ELISA.

|  | **Intra-assay CV (%)** | **Inter-assay CV (%)** |
| --- | --- | --- |
| CRP | 2.6 | 4.8 |
| sICAM-1 | 4.6 | 5.8 |
| sVCAM-1 | 2.5 | 4.8 |
| IFN-γ | 16.0 | 12.9 |
| IL-6 | 10.2 | 4.6 |
| IL-8 | 3.6 | 5.4 |
| IL-10 | 10.6 | 3.0 |
| TNF-α | 5.6 | 7.9 |
| suPAR | 3.0 | 7.5 |
| A2M | 3.9 | 19.2 |

**Intra-assay CV:** For CRP, sICAM-1, sVCAM-1, IFN-γ, IL-6, IL-8. IL-10 and TNF-α, intra-assay CV was calculated based on the mean CV of all participant sample concentration values for each marker (participant samples were measured in duplicate).

For suPAR and A2M, intra-assay CV was calculated based on the mean CV of absorbance values for standards comprising the standard curve (for these markers, each standard was measured in duplicate and participant samples were measured in singlet).

**Inter-assay CV:** For CRP, sICAM-1, sVCAM-1, interferon-γ, IL-6, IL-8. IL-10 and TNF-α, inter-assay CV was calculated based on the mean CV of concentration measurements for each standard comprising the standard curve on each plate.

For suPAR, inter-assay CV was calculated based on concentration measurements for the curve control on each plate.

For A2M, inter-assay CV was calculated based on the mean CV of absorbance values for standards comprising the standard curve on each plate.

Supplementary table 4. Coefficients of variation for PEA experiments.

|  | **Intra-assay CV (%)** | **Inter-assay CV (%)** |
| --- | --- | --- |
| Experiment 1 | 4 | 13 |
| Experiment 2 | 5 | 9 |

Intra- and inter-assay CV were calculated from the linear normalised protein expression values (NPX; an arbitrary unit used by Olink) of pooled plasma sample controls on each plate.

Supplementary table 5: Characteristics of the transdiagnostic clusters with missing data counts:

|  | | **Cluster 1**  **(n = 217)** | **Cluster 2**  **(n = 163)** | **P-value** | **Missing data, n (%)** |
| --- | --- | --- | --- | --- | --- |
| Psychotic disorder, n (%) | | 18 (8.3%) | 22 (13.5%) | 0.143 | 8 (2.1%) |
| Depressive disorder, n (%) | | 113 (52.1%) | 89 (54.6%) | 0.700 | <5 (<1.3%) |
| Generalised anxiety disorder, n (%) | | 157 (72.4%) | 111 (68.1%) | 0.432 | 0 (0%) |
| Sex | Male, n (%) | 58 (26.7%) | 39 (23.9%) | 0.616 | 0 (0%) |
|  | Female, n (%) | 159 (73.3%) | 124 (76.1%) |  |  |
| BMI, mean (SD) | | 24.6 (4.9) | 26.3 (7.0) | 0.049 | 9 (2.3%) |
| BMI >30 | No, n (%) | 189 (87.1%) | 125 (76.7%) | 0.012 | 9 (2.3%) |
|  | Yes, n (%) | 28 (12.9%) | 38 (23.3%) |  |  |
| Mean sample collection time (SD) | | 10:32 (1hr 47mins) | 10:37 (1hr 44mins) | 0.305 | 0 (0%) |
| Mean hours fasting (SD) | | 11.99 hrs (3.42 hrs) | 11.63 hrs (4.34 hrs) | 0.292 | 0 (0%) |
| Fasted less than 8 hours before blood sample collection | No, n (%) | 198 (91.2%) | 136 (83.4%) | 0.031 | 0 (0%) |
|  | Yes, n (%) | 19 (8.8%) | 27 (16.6%) |  |  |
| Daily smoker, n (%) | | 35 (16.1%) | 45 (27.6%) | 0.007 | <5 (<1.3%) |
| Major physical health condition, n (%) | | 5 (2.3%) | <5 (<3.1%) | 0.924 | 12 (3.2%) |
| Number of nights with sleep problems in past 7 nights, n (%) | 1-3 | 76 (35.0%) | 62 (38.0%) | 0.693 | <5 (<1.3%) |
|  | 4+ | 67 (30.9%) | 44 (27.0%) |  |  |
| Has received medication for hallucinations/delusions or other mental health problem, n (%) | | 39 (18.0%) | 42 (25.8%) | 0.087 | 8 (2.1%) |
| Suspected or definite psychotic experiences within the past year, n (%) | | 34 (15.7%) | 44 (27.0%) | 0.010 | 8 (2.1%) |
| Anhedonia within the past month | Less enjoyment than usual, n (%) | 144 (66.4%) | 98 (60.1%) | 0.106 | <5 (<1.3%) |
|  | Did not enjoy anything, n (%) | 8 (3.7%) | 14 (8.6%) |  |  |
| Not in employment, education or training | | 18 (8.3%) | 35 (21.5%) | <0.001 | 11 (2.8%) |

*Data supressed due to small cell counts.

Supplementary table 6: Difference in inflammatory marker means between clusters and controls, 95% CI. Difference in means of normalised inflammatory markers between cluster 1 and controls (left) and cluster 2 and controls (right) are presented in order of magnitude using controls as the reference group. The top 5 proteins with the greatest differences in magnitude between each cluster and controls are highlighted in bold.

| **Biomarker** | **Difference in means (Cluster 1 – Controls)** | **95% CI** | | **Biomarker** | **Difference in means (Cluster 2**  **– Controls)** | **95% CI** | |
| --- | --- | --- | --- | --- | --- | --- | --- |
| IL-6 | 0.05 | -0.10 | 0.20 | IFNy | 0.13 | -0.06 | 0.32 |
| IL6RA | -0.05 | -0.20 | 0.11 | IL-10 | 0.14 | -0.05 | 0.33 |
| A2M | -0.11 | -0.27 | 0.06 | IL1RT2 | 0.17 | 0.01 | 0.35 |
| IFNy | -0.13 | -0.28 | 0.00 | sVCAM-1 | 0.18 | 0.01 | 0.37 |
| IL-8 | -0.13 | -0.26 | 0.02 | IL-8 | 0.23 | 0.03 | 0.42 |
| CRP | -0.13 | -0.29 | 0.01 | IL6RA | 0.26 | 0.07 | 0.44 |
| suPAR | -0.14 | -0.29 | 0.00 | A2M | 0.29 | 0.12 | 0.46 |
| IL-10 | -0.15 | -0.30 | 0.00 | TNFa | 0.34 | 0.15 | 0.54 |
| sICAM-1 | -0.18 | -0.32 | -0.04 | IL1RT1 | 0.42 | 0.24 | 0.60 |
| TNFa | -0.24 | -0.40 | -0.08 | CRP | 0.47 | 0.28 | 0.66 |
| IL1RT1 | -0.32 | -0.47 | -0.17 | IL-6 | 0.49 | 0.31 | 0.67 |
| sVCAM-1 | -0.34 | -0.49 | -0.18 | CD93 | 0.50 | 0.34 | 0.66 |
| **IL1RT2** | -0.38 | -0.54 | -0.22 | **IL2RA** | 0.58 | 0.42 | 0.75 |
| **IL2RA** | -0.43 | -0.58 | -0.28 | **sICAM-1** | 0.61 | 0.42 | 0.80 |
| **TNFR1** | -0.44 | -0.58 | -0.31 | **TNFR2** | 0.81 | 0.66 | 0.96 |
| **CD93** | -0.53 | -0.68 | -0.39 | **TNFR1** | 0.87 | 0.71 | 1.03 |
| **TNFR2** | -0.66 | -0.78 | -0.53 | **suPAR** | 1.01 | 0.84 | 1.18 |

Supplementary table 7: Odds ratios from adjusted logistic regression analyses examining the association between inflammatory biomarker levels and cluster membership from the main analysis.

| **Biomarker** | **OR** | **95% CI** | |
| --- | --- | --- | --- |
| IFNy | 1.35 | 1.07 | 1.70 |
| IL-10 | 1.33 | 1.07 | 1.65 |
| IL-6 | 1.59 | 1.22 | 2.10 |
| IL-8 | 1.49 | 1.19 | 1.90 |
| TNFa | 1.74 | 1.39 | 2.21 |
| CRP | 1.91 | 1.47 | 2.51 |
| sICAM-1 | 2.43 | 1.87 | 3.21 |
| sVCAM-1 | 1.89 | 1.50 | 2.43 |
| suPAR | 5.25 | 3.64 | 7.89 |
| A2M | 1.57 | 1.25 | 1.99 |
| TNFR2 | 37.38 | 18.32 | 87.46 |
| TNFR1 | 15.43 | 9.13 | 28.14 |
| IL6RA | 1.32 | 1.04 | 1.67 |
| IL2RA | 4.21 | 3.03 | 6.04 |
| IL1RT2 | 1.95 | 1.54 | 2.51 |
| IL1RT1 | 2.88 | 2.19 | 3.88 |
| CD93 | 4.83 | 3.45 | 7.01 |

Models were adjusted for sex, BMI, daily smoking, medication use for mental health problems and NEET status. Interferon gamma (IFN-γ), interleukin-10 (IL-10), interleukin-6 (IL-6), interleukin-8 (IL-8), tumour necrosis factor alpha (TNF-α), C-reactive protein (CRP), soluble intracellular adhesion molecule-1s (ICAM-1), soluble vascular cell adhesion molecule-1 (sVCAM-1), soluble urokinase plasminogen activation receptor (suPAR), alpha-2-macroglobulin (A2M), tumour necrosis factor receptor 1 (TNF-R1), tumour necrosis factor receptor 2 (TNF-R2), interleukin-1 receptor type 1 (IL-1RT1), interleukin-1 receptor type 2 (IL-1RT2), interleukin-2 receptor subunit alpha (IL-2RA), interleukin-6 receptor subunit alpha (IL-6RA), cluster of differentiation 93 (CD93).

Supplementary table 8: The relationship between clinical variables and each standardised biomarker.

For each comparison where the difference in standardised means is given, the negative class is used as the reference group.

|  | **BMI** | | **Male Sex** | | **Daily Smoking** | | **Chronic Physical Health Condition** | | **Medication use for MH** | | **Psychosis** | | **NEET status** | |
| --- | --- | --- | --- | --- | --- | --- | --- | --- | --- | --- | --- | --- | --- | --- |
| Biomarker | Spearman's ρ | P-value | Difference in means | P-value | Difference in means | P-value | Difference in means | P-value | Difference in means | P-value | Difference in means | P-value | Difference in means | P-value |
| **IFNy** | 0.04 | 0.277 | 0.08 | 0.174 | -0.02 | 0.086 | 0.37 | 0.133 | 0.05 | 0.368 | 0.08 | 0.214 | 0.19 | 0.009 |
| **IL-10** | 0.02 | 0.567 | -0.02 | 0.162 | 0.21 | 0.057 | 0.45 | 0.057 | -0.01 | 0.498 | 0.10 | 0.175 | 0.02 | 0.225 |
| **IL-6** | 0.32 | 0.000 | -0.01 | 0.385 | 0.38 | 0.000 | 0.14 | 0.262 | 0.34 | 0.007 | 0.56 | 0.000 | 0.21 | 0.045 |
| **IL-8** | 0.07 | 0.065 | -0.27 | 0.000 | 0.15 | 0.007 | 0.42 | 0.233 | 0.15 | 0.049 | 0.09 | 0.286 | 0.10 | 0.287 |
| **TNFa** | 0.16 | 0.000 | -0.23 | 0.001 | 0.09 | 0.193 | 0.18 | 0.223 | 0.21 | 0.057 | 0.33 | 0.012 | 0.31 | 0.001 |
| **CRP** | 0.37 | 0.000 | 0.38 | 0.000 | 0.03 | 0.458 | 0.34 | 0.117 | 0.30 | 0.009 | 0.21 | 0.139 | 0.23 | 0.042 |
| **sICAM-1** | 0.12 | 0.000 | 0.03 | 0.376 | 0.40 | 0.000 | 0.22 | 0.476 | 0.23 | 0.049 | 0.42 | 0.007 | 0.08 | 0.339 |
| **sVCAM-1** | -0.03 | 0.356 | -0.20 | 0.001 | -0.12 | 0.301 | 0.20 | 0.187 | -0.04 | 0.249 | 0.08 | 0.383 | -0.06 | 0.137 |
| **suPAR** | 0.18 | 0.000 | 0.57 | 0.000 | 0.51 | 0.000 | 0.27 | 0.163 | 0.57 | 0.000 | 0.54 | 0.001 | 0.35 | 0.004 |
| **A2M** | -0.04 | 0.298 | 0.20 | 0.007 | -0.01 | 0.310 | -0.02 | 0.467 | 0.09 | 0.295 | 0.29 | 0.054 | 0.15 | 0.163 |
| **TNFR2** | 0.17 | 0.000 | -0.01 | 0.293 | 0.04 | 0.275 | 0.38 | 0.129 | 0.19 | 0.046 | 0.36 | 0.021 | 0.34 | 0.002 |
| **TNFR1** | 0.20 | 0.000 | -0.17 | 0.008 | 0.09 | 0.135 | 0.31 | 0.263 | 0.18 | 0.138 | 0.34 | 0.026 | 0.43 | 0.000 |
| **IL6RA** | 0.13 | 0.000 | -0.27 | 0.000 | 0.17 | 0.055 | 0.20 | 0.241 | 0.16 | 0.118 | 0.13 | 0.301 | 0.01 | 0.412 |
| **IL2RA** | 0.07 | 0.067 | 0.10 | 0.068 | 0.16 | 0.020 | 0.25 | 0.151 | 0.28 | 0.008 | 0.44 | 0.009 | 0.43 | 0.000 |
| **IL1RT2** | 0.04 | 0.242 | -0.57 | 0.000 | 0.03 | 0.342 | -0.14 | 0.144 | -0.19 | 0.049 | 0.03 | 0.364 | -0.05 | 0.400 |
| **IL1RT1** | -0.15 | 0.000 | 0.02 | 0.367 | 0.02 | 0.404 | 0.05 | 0.493 | -0.11 | 0.320 | -0.15 | 0.264 | 0.23 | 0.008 |
| **CD93** | -0.02 | 0.530 | -0.24 | 0.001 | 0.11 | 0.056 | -0.02 | 0.364 | -0.01 | 0.475 | 0.18 | 0.061 | 0.30 | 0.010 |

Supplementary figures:

**
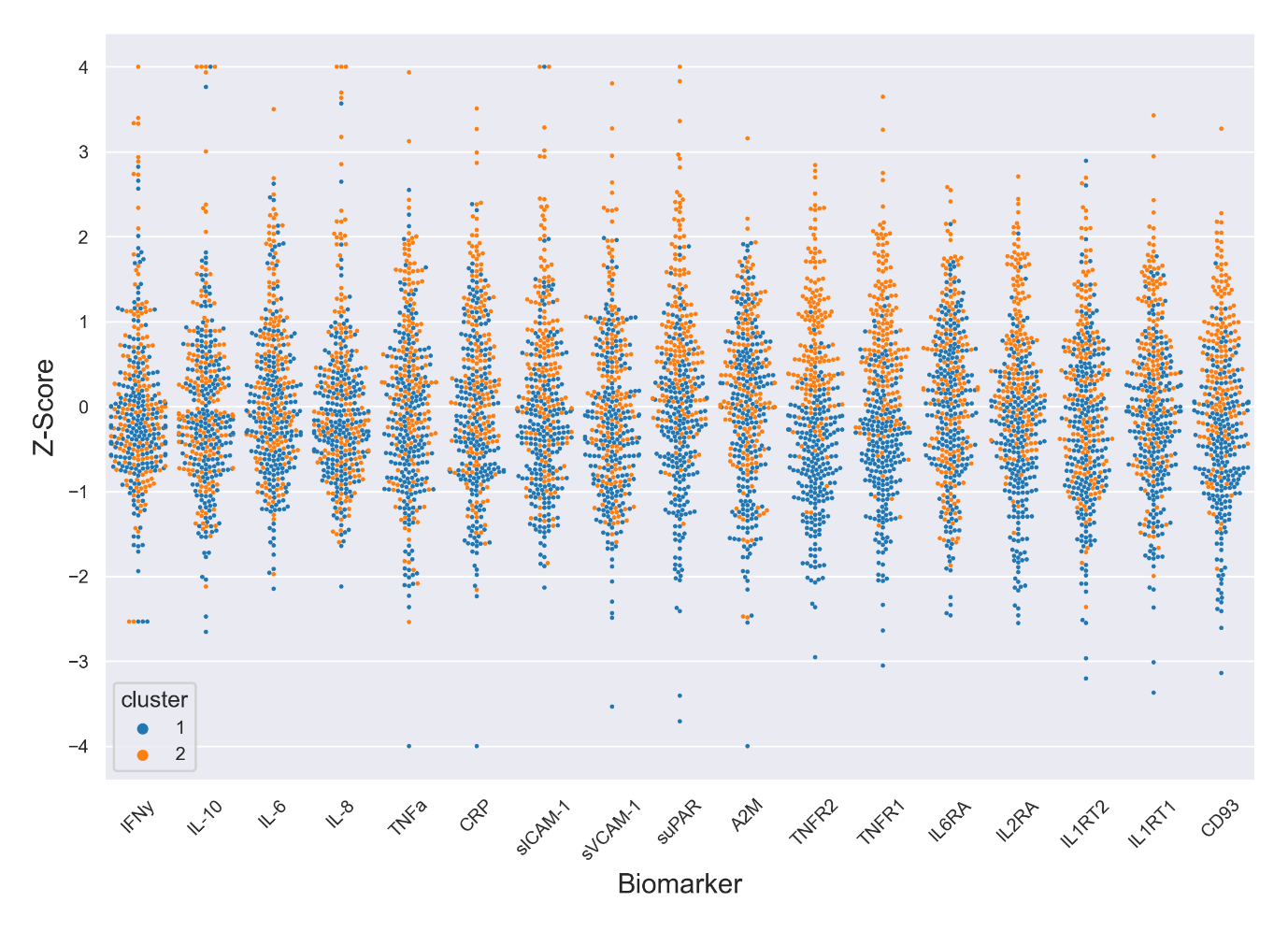
**

Supplementary figure 1: Distribution of standardised inflammatory biomarker values in each cluster as determined in the main analysis. A consensus clustering solution across 100 subsamples was determined by the algorithm HYDRA, adjusting for sex and BMI. Simulation with the Sigclust method indicated that the clusters explained the data better than a Gaussian distribution (*p* = 0.021). Interferon gamma (IFN-γ), interleukin-10 (IL-10), interleukin-6 (IL-6), interleukin-8 (IL-8), tumour necrosis factor alpha (TNF-α), C-reactive protein (CRP), soluble intracellular adhesion molecule-1s (ICAM-1), soluble vascular cell adhesion molecule-1 (sVCAM-1), soluble urokinase plasminogen activation receptor (suPAR), alpha-2-macroglobulin (A2M), tumour necrosis factor receptor 1 (TNF-R1), tumour necrosis factor receptor 2 (TNF-R2), interleukin-1 receptor type 1 (IL-1RT1), interleukin-1 receptor type 2 (IL-1RT2), interleukin-2 receptor subunit alpha (IL-2RA), interleukin-6 receptor subunit alpha (IL-6RA), cluster of differentiation 93 (CD93).

**
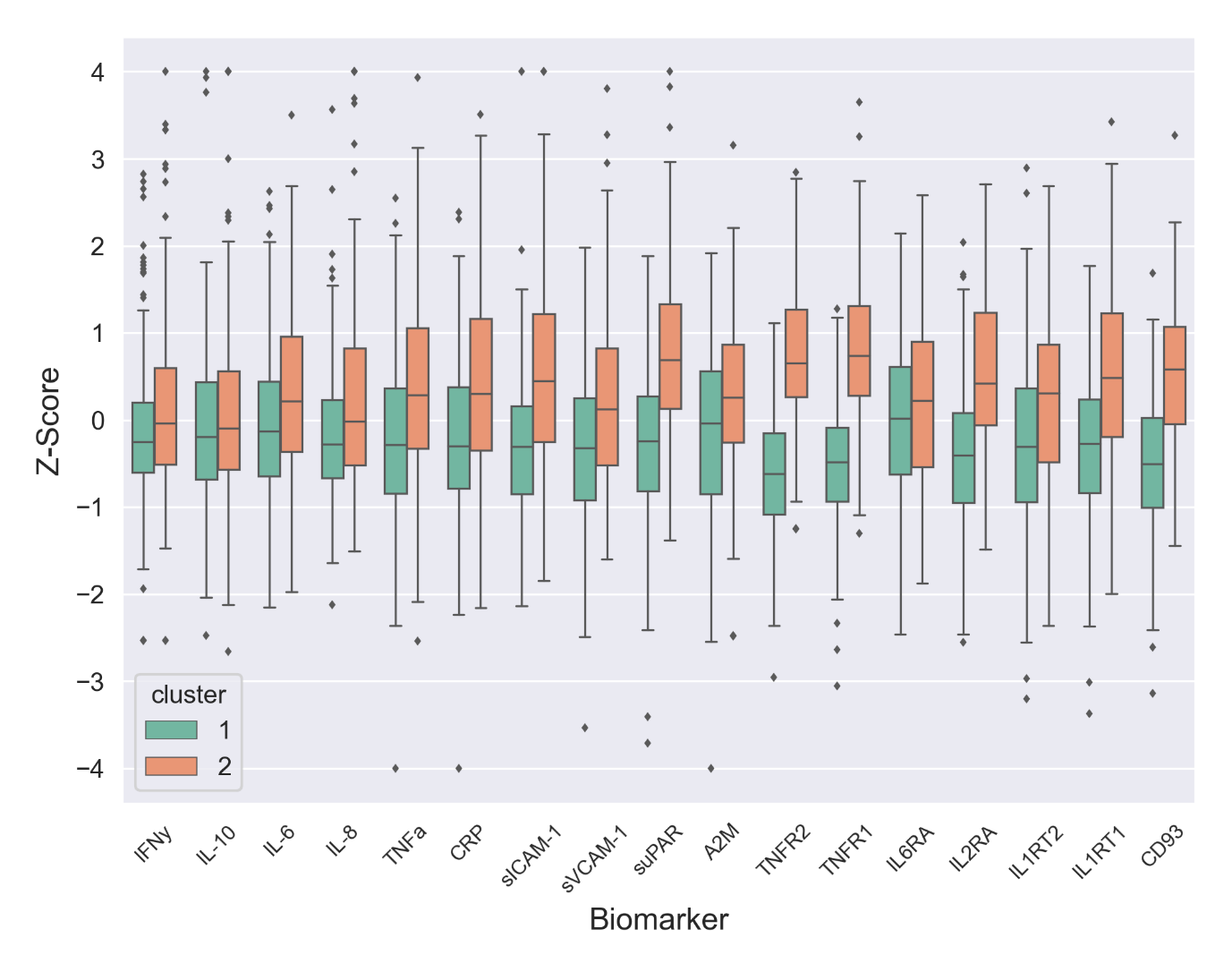
**

Supplementary figure 2: Distribution of standardised inflammatory biomarker values in each cluster as determined in the sensitivity analysis adjusting for daily smoking. A consensus clustering solution across 100 subsamples was determined by the algorithm HYDRA, adjusting for sex, BMI and daily smoking. Simulation with the Sigclust method indicated that the clusters explained the data better than a Gaussian distribution (*p* = 0.039). Interferon gamma (IFN-γ), interleukin-10 (IL-10), interleukin-6 (IL-6), interleukin-8 (IL-8), tumour necrosis factor alpha (TNF-α), C-reactive protein (CRP), soluble intracellular adhesion molecule-1s (ICAM-1), soluble vascular cell adhesion molecule-1 (sVCAM-1), soluble urokinase plasminogen activation receptor (suPAR), alpha-2-macroglobulin (A2M), tumour necrosis factor receptor 1 (TNF-R1), tumour necrosis factor receptor 2 (TNF-R2), interleukin-1 receptor type 1 (IL-1RT1), interleukin-1 receptor type 2 (IL-1RT2), interleukin-2 receptor subunit alpha (IL-2RA), interleukin-6 receptor subunit alpha (IL-6RA), cluster of differentiation 93 (CD93).

**
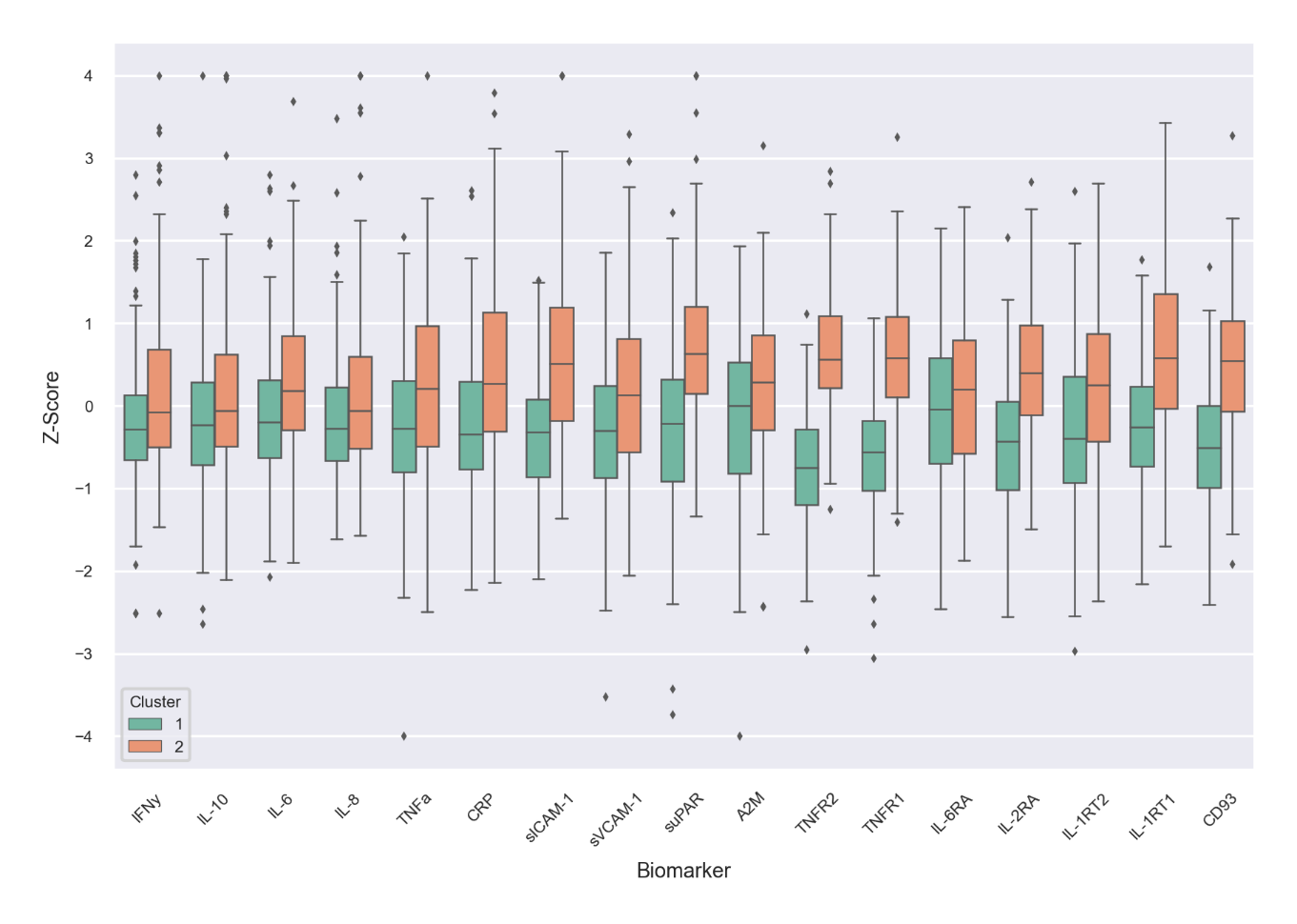
**

Supplementary figure 3: Distribution of standardised inflammatory biomarker values in each cluster as determined in the sensitivity analysis excluding individuals with chronic inflammatory disorders or BMI >30. A consensus clustering solution across 100 subsamples was determined by the algorithm HYDRA, adjusting for sex and BMI, excluding individuals with chronic inflammatory disorders or BMI >30. Simulation with the Sigclust method indicated that the clusters explained the data better than a Gaussian distribution (*p* = 0.017). Interferon gamma (IFN-γ), interleukin-10 (IL-10), interleukin-6 (IL-6), interleukin-8 (IL-8), tumour necrosis factor alpha (TNF-α), C-reactive protein (CRP), soluble intracellular adhesion molecule-1s (ICAM-1), soluble vascular cell adhesion molecule-1 (sVCAM-1), soluble urokinase plasminogen activation receptor (suPAR), alpha-2-macroglobulin (A2M), tumour necrosis factor receptor 1 (TNF-R1), tumour necrosis factor receptor 2 (TNF-R2), interleukin-1 receptor type 1 (IL-1RT1), interleukin-1 receptor type 2 (IL-1RT2), interleukin-2 receptor subunit alpha (IL-2RA), interleukin-6 receptor subunit alpha (IL-6RA), cluster of differentiation 93 (CD93).


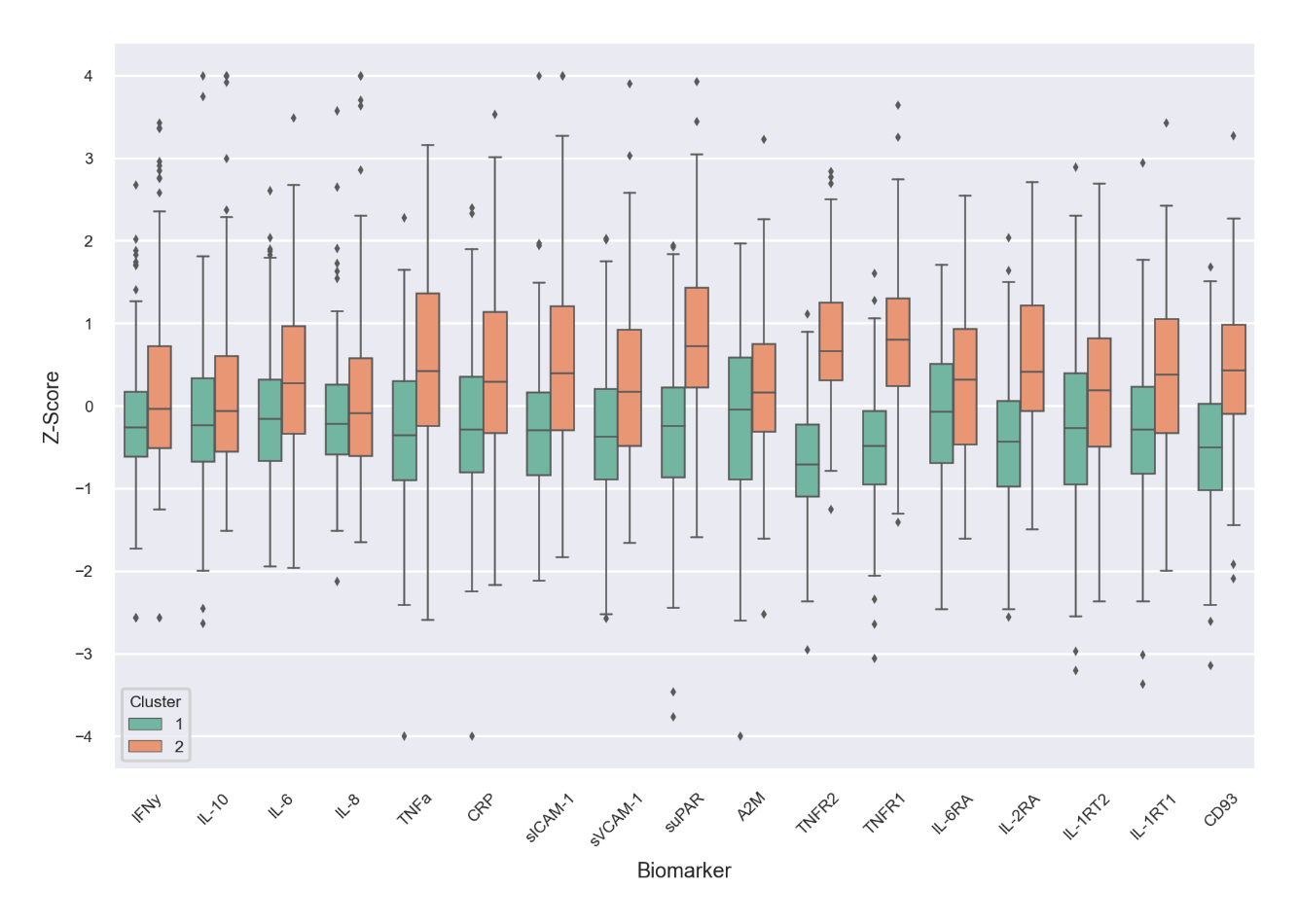


Supplementary figure 4: Distribution of standardised inflammatory biomarker values in each cluster as determined in the sensitivity analysis excluding individuals who fasted for less than 8 hours before their blood sample was taken. A consensus clustering solution across 100 subsamples was determined by the algorithm HYDRA, adjusting for sex and BMI excluding individuals who fasted for less than 8 hours before their blood sample was taken. Simulation with the Sigclust method indicated that the clusters explained the data better than a Gaussian distribution (*p* = 0.048). Interferon gamma (IFN-γ), interleukin-10 (IL-10), interleukin-6 (IL-6), interleukin-8 (IL-8), tumour necrosis factor alpha (TNF-α), C-reactive protein (CRP), soluble intracellular adhesion molecule-1s (ICAM-1), soluble vascular cell adhesion molecule-1 (sVCAM-1), soluble urokinase plasminogen activation receptor (suPAR), alpha-2-macroglobulin (A2M), tumour necrosis factor receptor 1 (TNF-R1), tumour necrosis factor receptor 2 (TNF-R2), interleukin-1 receptor type 1 (IL-1RT1), interleukin-1 receptor type 2 (IL-1RT2), interleukin-2 receptor subunit alpha (IL-2RA), interleukin-6 receptor subunit alpha (IL-6RA), cluster of differentiation 93 (CD93).


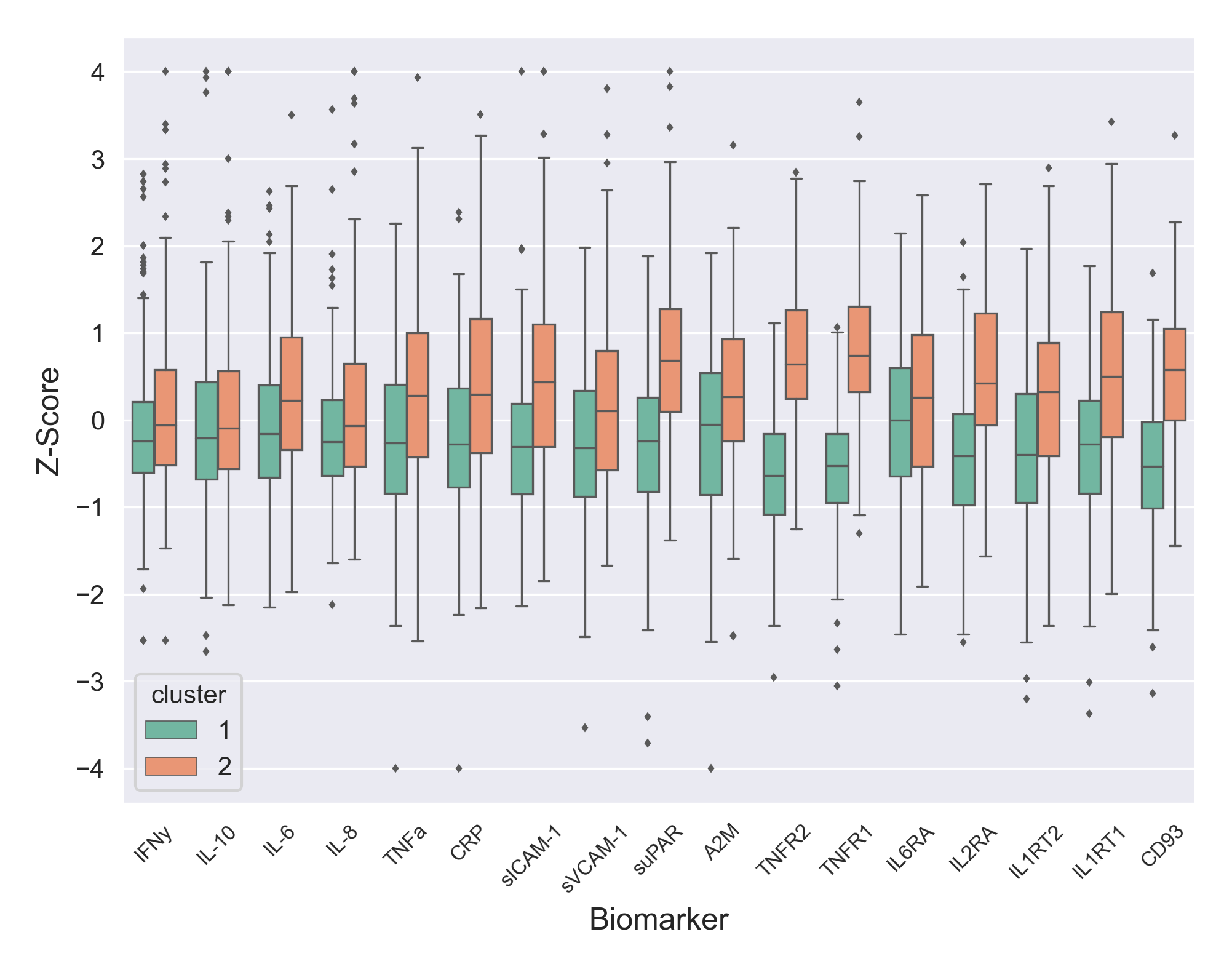


Supplementary figure 5: Distribution of standardised inflammatory biomarker values in each cluster as determined in the sensitivity analysis adjusting for PEA experiment. A consensus clustering solution across 100 subsamples was determined by the algorithm HYDRA, adjusting for sex, BMI and PEA experiment. Simulation with the Sigclust method indicated that the clusters explained the data better than a Gaussian distribution (*p* = 0.038). Interferon gamma (IFN-γ), interleukin-10 (IL-10), interleukin-6 (IL-6), interleukin-8 (IL-8), tumour necrosis factor alpha (TNF-α), C-reactive protein (CRP), soluble intracellular adhesion molecule-1s (ICAM-1), soluble vascular cell adhesion molecule-1 (sVCAM-1), soluble urokinase plasminogen activation receptor (suPAR), alpha-2-macroglobulin (A2M), tumour necrosis factor receptor 1 (TNF-R1), tumour necrosis factor receptor 2 (TNF-R2), interleukin-1 receptor type 1 (IL-1RT1), interleukin-1 receptor type 2 (IL-1RT2), interleukin-2 receptor subunit alpha (IL-2RA), interleukin-6 receptor subunit alpha (IL-6RA), cluster of differentiation 93 (CD93).

**
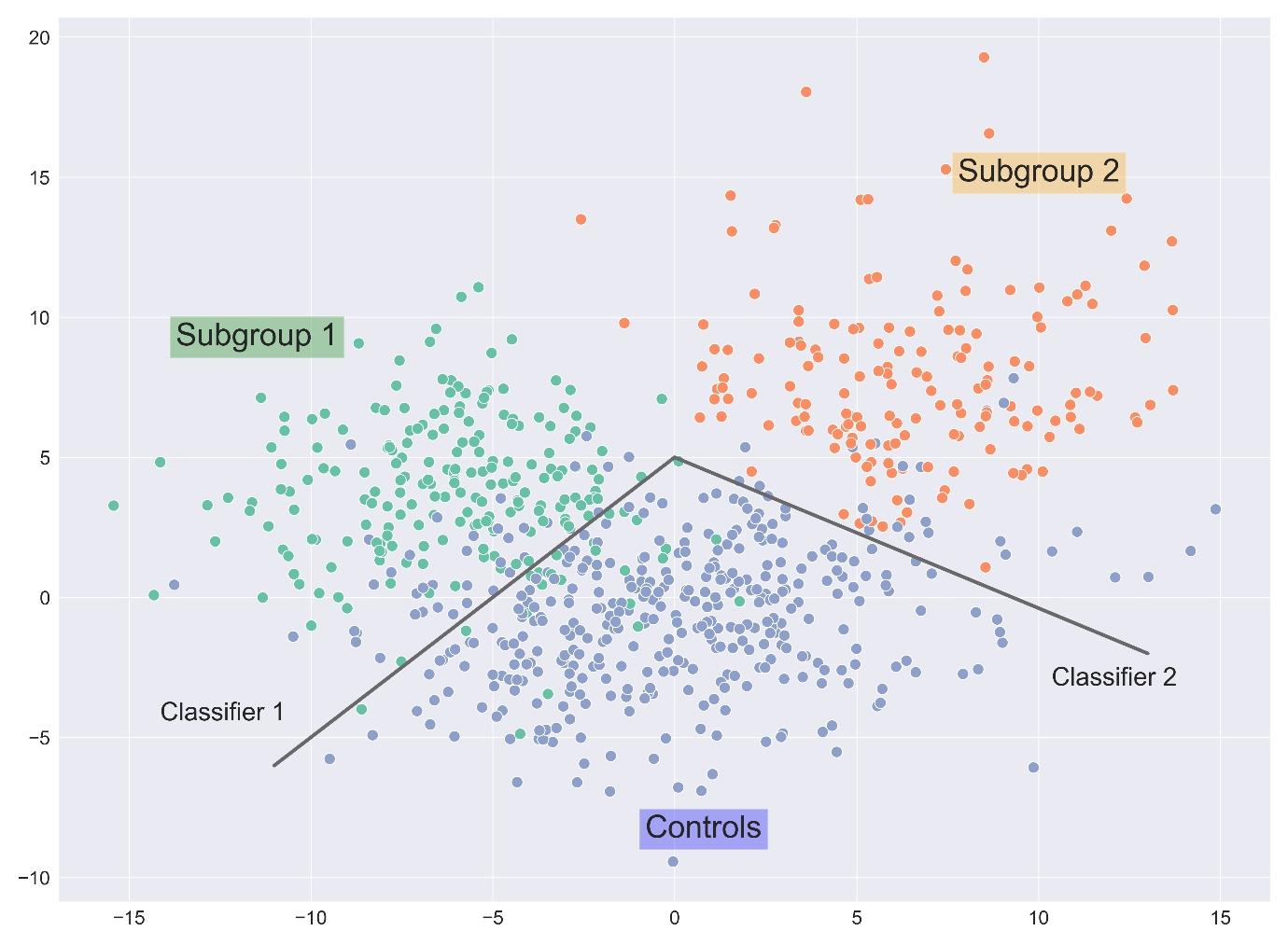
**

Supplementary figure 6: Depiction of semi-supervised clustering with HYDRA. HYDRA^1^ separates cases from healthy controls by fitting multiple linear maximum-margin classifiers or hyperplanes. Together, these hyperplanes form a shape called a convex polytope which divides cases and controls. Patient subtypes are derived from the association of groups of cases with individual hyperplanes. Data points plotted do not represent data points from this study and are for illustrative purposes only.

Supplementary results:

**Cluster analysis**

In logistic regression models, there was an association between each inflammatory biomarker level and cluster membership while adjusting for sex, BMI, daily smoking, medication use for mental health problems and NEET status (Supplementary Table 7).

In a logistic regression model, the association between NEET status and cluster membership designation from the main analysis remained while adjusting for sex, BMI, daily smoking, medication use for mental health problems, whether individuals fasted for <8 hours and PEA experiment number (OR 2.92, 95% CI 1.55, 5.65)

**Sensitivity analyses**

We conducted a sensitivity analysis where the algorithm HYDRA was run excluding individuals who did not fast at least 8 hours before their blood sample was taken. The highest ARI (0.51, SD = 0.02) was for a two-cluster solution. Simulation with the Sigclust method indicated that the clusters explained the data better than a Gaussian distribution (*p* = 0.046). The inflammatory marker distributions of the clusters obtained in this sensitivity analysis was in agreement with our main analysis (Supplementary figure 4).

We conducted a sensitivity analysis where the algorithm HYDRA was run with PEA experiment number as an additional covariate. The highest ARI (0.48) was for a two-cluster solution. Simulation with the Sigclust method indicated that the clusters explained the data better than a Gaussian distribution (*p* = 0.038). The inflammatory marker distributions of the clusters obtained in this sensitivity analysis was in agreement with our main analysis (Supplementary figure 5).

References

1. Varol E, Sotiras A, Davatzikos C, Alzheimer’s Disease Neuroimaging Initiative. HYDRA: Revealing heterogeneity of imaging and genetic patterns through a multiple max-margin discriminative analysis framework. Neuroimage. 2017;145:346–364.

2. Hubert L, Arabie P. Comparing partitions. Journal of Classification. 1985;2:193–218.

3. Chand GB, Dwyer DB, Erus G, Sotiras A, Varol E, Srinivasan D, et al. Two distinct neuroanatomical subtypes of schizophrenia revealed using machine learning. Brain. 2020;143:1027–1038.
